# Antiviral Efficacy and Safety of Molnupiravir Against Omicron Variant Infection: A Randomized Controlled Clinical Trial

**DOI:** 10.3389/fphar.2022.939573

**Published:** 2022-06-15

**Authors:** Rongrong Zou, Ling Peng, Dan Shu, Lei Zhao, Jianfeng Lan, Guoyu Tan, Jinghan Peng, Xiangyi Yang, Miaona Liu, Chenhui Zhang, Jing Yuan, Huxiang Wang, Song Li, Hongzhou Lu, Wu Zhong, Yingxia Liu

**Affiliations:** ^1^ Shenzhen Key Laboratory of Pathogen and Immunity, National Clinical Research Center for Infectious Disease, State Key Discipline of Infectious Disease, Shenzhen Third People’s Hospital, Second Hospital Affiliated to Southern University of Science and Technology, Shenzhen, China; ^2^ National Engineering Research Center for the Emergency Drug, Beijing Institute of Pharmacology and Toxicology, Beijing, China

**Keywords:** Omicron variant, SARS-CoV-2, molnupiravir, antiviral efficacy, clinical trial

## Abstract

**Background:** The rapid worldwide spread of the Omicron variant of SARS-CoV-2 has unleashed a new wave of COVID-19 outbreaks. The efficacy of molnupiravir, an approved drug, is still unknown in patients infected with the Omicron variant.

**Objective:** Evaluated the antiviral efficacy and safety of molnupiravir in patients infected with SARS-CoV-2 Omicron variant, with symptom duration within 5 days.

**Methods:** We conducted a randomized, controlled trial involving patients with mild or moderate COVID-19. Patients were randomized to orally receive molnupiravir (800 mg) plus basic treatment or only basic treatment for 5 days (BID). The antiviral efficacy of the drug was evaluated using reverse transcriptase polymerase chain reaction.

**Results:** Results showed that the time of viral RNA clearance (primary endpoint) was significantly decreased in the molnupiravir group (median, 9 days) compared to the control group (median, 10 days) (Log-Rank *p* = 0.0092). Of patients receiving molnupiravir, 18.42% achieved viral RNA clearance on day 5 of treatment, compared to the control group (0%) (*p* = 0.0092). On day 7, 40.79%, and 6.45% of patients in the molnupiravir and control groups, respectively, achieved viral RNA clearance (*p* = 0.0004). In addition, molnupiravir has a good safety profile, and no serious adverse events were reported.

**Conclusion:** Molnupiravir significantly accelerated the SARS-CoV-2 Omicron RNA clearance in patients with COVID-19.

**Clinical Trial Registration:** [chictr.org.cn], identifier [ChiCTR2200056817].

## Introduction

The Omicron variant of severe acute respiratory syndrome coronavirus 2 (SARS-CoV-2) has rapidly replaced the Delta variant as the dominating SARS-CoV-2 variant, which has higher infectivity and stronger vaccine breakthrough ability (Chen et al., 2022; Saxena et al., 2022). Among the three lineages of Omicron: BA.1 (B.1.1.529.1), BA.2 (B.1.1.529.2), and BA.3 (B.1.1.529.3), BA.2 is rapidly increasing worldwide since January 2022 and has become a new dominating “variant of concern” in China (Cheng et al., 2022; World Health Organization, 2022). With the current surge of Omicron infections in China, both the absolute number of patients hospitalized with coronavirus disease 2019 (COVID-19) and the percentage of total COVID-19 hospitalizations have been increasing recently. Although the direct effects of SARS-CoV-2 Omicron variant on patients have been largely mild, the impact on people’s health is substantial due to the large number of infected individuals and potential virus-induced lung injury (He et al., 2021). Thus, an oral, effective, and direct-acting therapeutic is needed to block the transmission of SARS-CoV-2 Omicron variant.

Molnupiravir, a small-molecule drug, has been reported to have shown good anti-SARS-CoV-2 efficacy in phase II/III clinical trials (Jayk Bernal et al., 2021; Fischer et al., 2022). It has been approved by the United Kingdom MHRA and has received an Emergency Use Authorization from the FDA for the treatment of COVID-19. Recent studies have shown that molnupiravir could inhibits Omicron variant *in vitro* and *in vivo* (Rosenke et al., 2022; Takashita et al., 2022; Uraki et al., 2022). However, data on its efficacy and safety in the population infected with the Omicron variant is still lacking.

In this study, we performed a randomized, controlled study to evaluate the clinical efficacy of molnupiravir for COVID-19 patients infected with Omicron BA.2 at the Third People’s Hospital of Shenzhen. We endeavored to compare the antiviral efficacy between patients who were treated with molnupiravir plus basic treatment and those treated only with basic treatment. This study will provide practical insight for the treatment against SARS-CoV-2 Omicron variant infections.

## Methods

### Study Design

We conducted a randomized, controlled trial of molnupiravir in patients infected with Omicron variant (ChiCTR2200056817, chictr.org.cn). The protocol was reviewed and approved by the Medical Ethics Committee of the Third People’s Hospital of Shenzhen, and the trial was conducted in accordance with the Declaration of Helsinki and Good Clinical Practice guidelines of the International Conference on Harmonization. Molnupiravir was provided by HUAHAI Pharmaceutical.

From March 3 to 21 March 2022, patients with confirmed mild/moderate COVID-19 were admitted and enrolled at the Third People’s Hospital of Shenzhen. Eligible patients include male or nonpregnant females aged ≥18 years and ≤80 years, who were tested positive for SARS-CoV-2 Omicron variant and had initial onset of symptoms for ≤5 days prior to the day of treatment. Exclusion criteria included severe vomiting or intolerance of oral drugs for other reasons, pregnancy or lactation, patients retesting positive for COVID-19, and those who had received antibody therapy, plasma therapy, or other investigational drugs for SARS-CoV-2. All the patients provided a written informed consent.

Patients were randomly grouped (2:1) into molnupiravir (*n* = 80) and control groups (*n* = 36) using a random number table. Molnupiravir (800 mg twice per day) were administered orally for 5 days in the hospital. In addition, all patients received the same basic treatment, which consisted of vitamin C, lianhuaqingwen granule, and nasal irrigation. Pharyngeal swab was collected every other day to determine the antiviral effect of molnupiravir against SARS-CoV-2 using RT-PCR. Adverse events were monitored throughout the study period.

### Antiviral Efficacy

The primary endpoint was the time of viral RNA clearance measured using RT-PCR analysis of pharyngeal swab. The time of viral RNA clearance was defined as the number of days it took to have a negative viral RNA test result post-randomization (two consecutive times or 24 h at two intervals). Samples were tested for detection of ORF1ab and N genes. A negative RT-PCR result was confirmed only when all test results were reported negative (Ct > 35). The secondary antiviral efficacy endpoint included the percentage of patients who were negative for SARS-CoV-2 infectious virus on days 5, 7, and 10.

### Statistical Analyses

The measurement data conforming to non-normal distribution were exhibited as median, and the Wilcoxon rank-sum test was used for comparison between groups. Meanwhile, the count data were exhibited as cases (%), and comparisons between groups were made using the chi-square test. A *p* value <0.05 was considered statistically significant. Time of viral RNA clearance was summarized using the Kaplan-Meier methodology, and log-rank test was used for comparison of the efficacy between treatments. The percentage of patients who were negative for viral RNA was summarized and compared between groups using Fisher’s exact test.

## Results

### Patient Demographics and Clinical Characteristics

**FIGURE 1:**
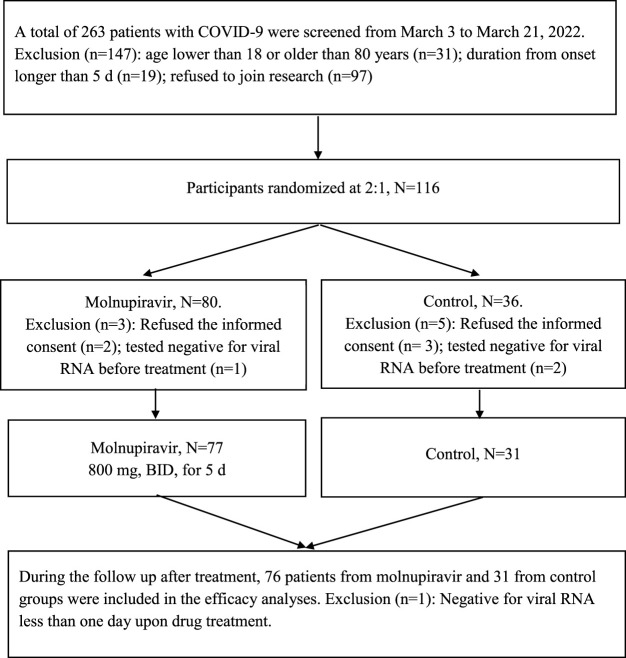
CONSORT diagram for this trial.

From March 3 to 21 March 2022, 116 patients were randomly grouped, with 108 receiving molnupiravir or only basic treatment (Figure 1). The patients randomized to the control and molnupiravir groups were well matched. Baseline characteristics were generally similar across the groups, and baseline laboratory test results were well balanced (Table 1).

**TABLE 1:** Patient demographics and clinical characteristics at baseline.

Characteristics	Molnupiravir (*n* = 77)	Control (*n* = 31)	*p* value
Median age (range)	39 (20, 63)	42 (22, 61)	0.363
Male	43/77 (55.8%)	17/31 (54.8%)	0.924
Median BMI (IQR)	23.44 (21.17, 26.04)	23.34 (21.45, 25.65)	0.873
Presenting symptoms			
Fever	32/77 (41.6%)	9/31 (29.0%)	0.225
Maximum body temperature, C (mean, range)	38.6 (37.8, 39.5)	38.2 (37.5, 39)	0.061
Two or more symptoms^a^	59/77 (76.6%)	24/31 (77.4%)	0.929
One underlying disease^#^	15/77 (19.5%)	5/31 (16.1%)	0.685
Two or more underlying disease	3/77 (3.9%)	2/31 (6.5%)	0.567
Disease severity			
Severe	0	0	
Moderate	3/77 (3.9%)	1/31 (3.2%)	1.000
Mild	74/77 (96.1%)	30/31 (96.8%)	1.000
Virus nucleic acid			
ORF gene, median (IQR)	18.35 (16.78, 22.32)	18.19 (16.63, 22.44)	0.638
N gene, median (IQR)	17.98 (15.68, 21.24)	17.51 (15.13, 21.43)	0.865
Vaccination			
Two doses	22/77 (28.6%)	11/31 (35.5%)	0.833
Three doses	48/77 (62.3%)	18/31 (58.1%)	0.851
Laboratory results			
WBC (× 10^9^/L)	5.37 (4.43, 7.14)	5.88 (4.53, 7.13)	0.543
LYM (× 10^9^/L)	1.21 (0.85, 1.72)	1.22 (0.88, 1.55)	0.760
HB (g/L)			
AST (U/L)	29 (22.9, 33.35)	30.2 (34.1, 25.2)	0.852
ALT (U/L)	22 (14.5, 33.5)	23 (14, 31)	0.794
CK (U/L)	76 (49, 112.5)	70 (54.5, 116.75)	0.981
CK-MB (ng/ml)	0.219 (0.219, 0.410)	0.219 (0.219, 0.250)	0.424
LDH (U/L)	168 (147.5, 187)	178 (154, 197)	0.288
IL-6 (pg/ml)	4.6 (2.34, 8.56)	7.30 (3.27, 12.18)	0.059
CRP (mg/L)	7.35 (2.80, 11.66)	7.00 (3.28, 15.77)	0.508
Bun (mmol/L)	3.86 (3.34, 4.75)	4.43 (3.26, 5.05)	0.347
Cr (μmol/L)	73.6 (59.85, 94)	72.1 (56.1, 83.45)	0.288
CD4 cells (count/μL)	447.5 (310.75, 611.75)	537 (328.5, 664.5)	0.336
CD8 cells (count/μL)	299.5 (173, 416.25)	363 (236, 507)	0.232
Abnormal CT scan features	3/77 (3.9%)	1/31 (3.2%)	1.000

a Cough, fatigue, loss of taste or smell, nasal congestion, runny nose, sore throat, myalgia, diarrhea, headache, chill, nausea or vomiting, dizziness, chest pain.

#Diabetes, hypertension, coronary heart disease, chronic liver disease, chronic kidney disease, chronic obstructive pulmonary disease, tumor.

IQR: interquartile range.

### Antiviral Efficacy

**FIGURE 2:**
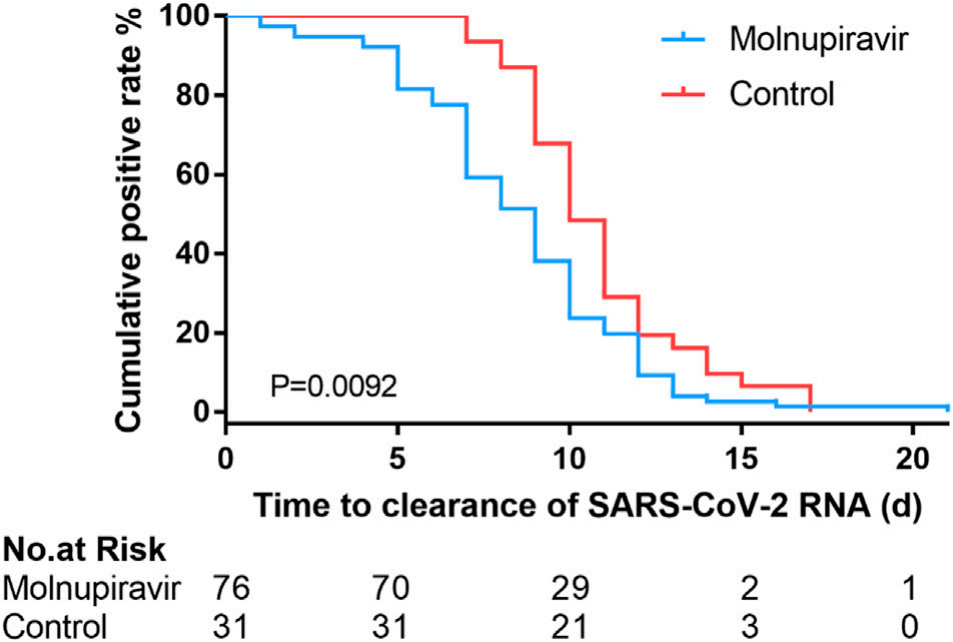
Kaplan-Meier plot showing the time of viral RNA clearance in the molnupiravir and control groups.

A total of 107 patients in the cohort were included for the primary efficacy analysis. One patient in the molnupiravir group was excluded from the antiviral efficacy analysis because of a negative result in the viral RNA test less than 12 h after taking the medicine. The primary outcome of this study was the time of viral RNA clearance. The median time of viral clearance were 9 days (95% CI: 7–9 days) and 10 days (95% CI: 9–11 days) in the molnupiravir and control groups, respectively. The median time was significantly reduced in the molnupiravir group (log-rank *p* = 0.0092, Figure 2 and Table 2). This result indicates that molnupiravir can effectively accelerate viral RNA clearance in patients infected with the Omicron variant, which is consistent with the antiviral efficacy of molnupiravir in treatment of COVID-19 (Fischer et al., 2022).

**TABLE 2:** Antiviral efficacy of molnupiravir.

Characteristics	Molnupiravir (*n* = 76)	Control (*n* = 31)	*p* value
Patients negative for viral RNA on day 5, *n* (%)	14(18.42)	0(0)	0.0092
Patients negative for viral RNA on day 7, *n* (%)	31(40.79)	2(6.45)	0.0004
Patients negative for viral RNA on day 10, *n* (%)	58(76.32)	16(51.61)	0.02
Median time for a negative viral RNA, days (95% CI)	9(7–9)	10(9–11)	0.0092

The key secondary virological endpoint of this trial was the percentage of patients who were negative for viral RNA on days 5, 7, and 10 (Table 2). On day 5 of treatment, the percentage of patients who were negative for viral RNA was 18.42% in the molnupiravir group and 0% in the control group (*p* = 0.0092). The percentage of patients who were negative for viral RNA also increased to 40.79% on day 7 in the molnupiravir group compared to the control group (6.45%) (*p* = 0.0004). The percentage of patients negative for viral RNA remained significantly different between molnupiravir and control groups on day 10 of treatment (molnupiravir group: 76.32%; control group: 51.61%) (*p* = 0.02).

### Safety

Three adverse events were reported in the molnupiravir group (Table 3). One patient (1.3%) had skin rash, and two patients (2.6%) had elevated levels of alanine transaminase. No grade 3 or higher adverse events were reported, and no adverse events led to the discontinuation of molnupiravir treatment.

**TABLE 3:** Summary of adverse events in the two groups.

Characteristic	Molnupiravir (*n* = 77)	Control (*n* = 31)	*p* value
Adverse events			
Rash	1 (1.3%)	0 (0%)	1.000
Elevated ALT	2 (2.6%)	0 (0%)	1.000

### Clinical Outcomes

The duration of fever, time of symptom alleviation and laboratory test results were collected to evaluate the overall health condition associated with COVID-19. At baseline, patients in the molnupiravir and control groups matched well in all presentation indicators, with no significant difference. In the laboratory test before discharge, all test values of patients recovered to the normal range, and no significant difference was observed between the groups (Table 4). The median time to alleviate COVID-19 symptoms between the molnupiravir and control groups was not statistically significant (5 days vs. 7 days, *p* = 0.499), and the results were consistent with previously published studies (Fischer et al., 2022). In addition, the median time of fever duration was not statistically between the two groups, although the duration of fever was reduced by 2 days in the molnupiravir group compared to the control group (1 day vs. 3 days, *p* = 0.096).

**TABLE 4:** Clinical outcomes associated with COVID-19.

Characteristics	Molnupiravir (*n* = 77)	Control (*n* = 31)	*p* value
Fever duration, days (median, IQR)	1 (1, 2)	3 (1, 3)	0.096
Symptoms alleviating time, days (median, IQR)	5 (3.75, 7)	7 (3, 7)	0.499
Laboratory test before discharge			
AST (U/L)	22.9 (21.4, 30.2)	25.5 (20.2, 28.7)	0.630
ALT (U/L)	21 (12.5, 36.5)	19 (13, 26)	0.847
CK (U/L)	43 (31, 73)	37 (31, 69)	0.425
CK-MB (ng/ml)	0.219 (0.291, 0.360)	0.219 (0.219, 0.3)	0.543
LDH (U/L)	168 (136, 181)	156 (147, 174)	0.917
IL-6 (pg/ml)	1.49 (1.49, 1.69)	1.57 (1.49, 3.28)	0.145
CRP (mg/L)	1.46 (0.66, 3.11)	1.00 (0.33, 6.79)	0.975
Bun (mmol/L)	4.63 (3.75, 5.01)	4.13 (3.34, 5.21)	0.555
Cr (μmol/L)	62 (56.3, 89.6)	67.5 (52.8, 72.1)	0.555

## Discussion

As of 22 March 2022, 470,839,745 confirmed COVID-19 cases were recorded, including 6,092,933 deaths, and a total of 10,925,055,390 doses of vaccine had been administered worldwide. The emerging Omicron variant of SARS-CoV-2 is highly infectious and has a stronger vaccine breakthrough ability against antibodies provided by existing vaccines, thus it is the dominating SARS-CoV-2 variant globally (Arora et al., 2022). The current effective tools to fight COVID-19 outbreaks are vaccination and small-molecule oral drug therapy. The main role of marketed COVID-19 vaccines is to reduce symptomatic infection and prevent severe illness. Unfortunately, no clinical trial on small-molecule oral medicine as treatment against COVID-19 caused by Omicron variant is available. Molnupiravir has been approved for marketing in the United Kingdom for the treatment of adults with mild to moderate COVID-19 and has been granted emergency use authorization from FDA (Food and Drug Administration, 2021; Merck, 2021).

In our study, we found that molnupiravir was effective in treating COVID-19 caused by Omicron variant infection, with few adverse effects. Compared to basic treatment, molnupiravir treatment significantly reduced viral load in pharyngeal swabs and accelerated viral RNA clearance in patients with COVID-19. In the completed phase 2A trial of molnupiravir, the median time of viral RNA clearance was 14 days in the molnupiravir group and 15 days in the control group (*p* = 0.013) (Fischer et al., 2022). In this study, the median time of viral RNA clearance in the molnupiravir group and the control group were 9 days and 10 days, respectively (*p* = 0.0092). This difference may be due to two possible reasons, as follows: 1) reduced replication capacity and pathogenicity of the Omicron variant compared to the other variants (Shuai et al., 2022), and 2) more than 90% of the patients enrolled in this trial received at least two doses of COVID-19 vaccine (molnupiravir group: 90.9%; control group: 93.6%). In the vaccination population, most of the patients received the inactivated vaccine (Sinavac), only 7.1 and 6.9% patients received other vaccines (BNT162b2 or Zifivax) in molnupiravir and control groups, respectively (Supplmentary Table S1). We have checked the neutralization antibody titers for all the vaccination patients and found that there was no significant difference on neutralization antibody titer between two groups (*p* = 0.521) (Supplementary Table S2). In term of T cell immunity, there was no significant difference in T cells (CD4 and CD8 cells) counts, as shown in Table 1. These results indicate that molnupiravir administration based on widespread vaccination may be more effective in responding to outbreaks caused by the Omicron variant.

Based on the higher infectivity and reduced pathogenicity of the Omicron variant, it is more likely to result in a great deal of patients with mild COVID-19 or asymptomatic infections, rather than severe COVID-19. As a result, the clearance of viral RNA from patients and interruption of viral transmission are more relevant in the fight against Omicron variant infections. This clinical trial was outlined to assess the efficacy and safety of molnupiravir against the Omicron variant of SARS-CoV-2 and did not focus on evaluating clinical endpoints, such as symptom duration or length of hospitalization. Furthermore, recruitment was not limited to patients with risk factors of progressing to severe disease. Patients did not benefit from molnupiravir treatment in terms of duration of fever and symptoms alleviating time during the observation period. Another limitation is that asymptomatic individuals were not recruited into the trial. In this population, which was positive for viral RNA but has no characteristic symptoms, treatment with molnupiravir that exert a direct antiviral effect is equally relevant and may be more effective.

This study provides biological evidence to support the use of molnupiravir in accelerating viral RNA clearance in patients infected with the Omicron variant of SARS-CoV-2, potentially reducing viruses early transmission. The dynamic zero COVID-19 strategy is the most effective prevention and control strategy proposed and implemented in China during the COVID-19 epidemic. In the face of a new wave of COVID-19 outbreaks caused by the Omicron variant, small-molecule oral drug therapy will contribute to the implementation of the dynamic zero COVID-19 strategy and accelerate the end of the epidemic.

## Data Availability

The raw data supporting the conclusions of this article will be made available by the authors, without undue reservation.
