# Supplementary material for: Antiviral Efficacy and Safety of Molnupiravir Against Omicron Variant Infection: A Randomized Controlled Clinical Trial
Source: Front Pharmacol. 2022 Jun 15;13:939573. doi: 10.3389/fphar.2022.939573 (PMC9248931; doi:10.3389/fphar.2022.939573)
Supplement: Supplementary file 1 [file DataSheet1.docx]

| **Supplemental Data**   \| Table S1 Vaccination status \| \| --- \| |
| --- | --- |

|  | Molnupiravir | | Control | |
| --- | --- | --- | --- | --- |
|  | Two doses,  N=22 | Three doses,  N=48 | Two doses,  N=11 | Three doses,  N =18 |
| Inactivated vaccine | 20 | 45 | 11 | 16 |
| 2 Inactivated vaccine +1 BNT162b2 |  | 2 |  | 2 |
| 2 BNT162b2 | 2 |  |  |  |
| 3 Zifivax |  | 1 |  |  |

| Table S2 Neutralization antibody titer |
| --- |

|  | SARS-CoV-IgG*, median (IQR) |
| --- | --- |
| Molnupiravir  (N = 70) | 66.26 (116.49) |
| Control  (N = 29) | 39.91 (197.68) |
| P value | 0.521 (rank sum test: z=-0.642) |
